# Tolerability and Efficacy of Customized IncobotulinumtoxinA Injections for Essential Tremor: A Randomized, Double-Blind, Placebo-Controlled Study

**DOI:** 10.3390/toxins12120807

**Published:** 2020-12-20

**Authors:** Mandar Jog, Jack Lee, Astrid Scheschonka, Robert Chen, Farooq Ismail, Chris Boulias, Douglas Hobson, David King, Michael Althaus, Olivier Simon, Hanna Dersch, Steven Frucht, David M. Simpson

**Affiliations:** 1 Lawson Health Research Institute, London, ON N6C2R5, Canada; jack.lee@lhsc.on.ca; 2 MDDT Inc., London, ON N6G0J3, Canada; 3 Merz Pharmaceuticals GmbH, 60318 Frankfurt am Main, Germany; Astrid.Scheschonka@merz.de (A.S.); Michael.Althaus@merz.de (M.A.); simonolivier77@gmail.com (O.S.); Hanna.Dersch@merz.de (H.D.); 4 Krembil Brain Institute, University Health Network, University of Toronto, Toronto, ON M5T1M8, Canada; Robert.Chen@uhn.ca; 5 West Park Healthcare Center, University of Toronto, Toronto, ON M6M2J5, Canada; Farooq.Ismail@westpark.org (F.I.); Chris.Boulias@westpark.org (C.B.); 6 Department of Neurology, University of Manitoba, Winnipeg, MB R3T2N2, Canada; Douglas.Hobson@umanitoba.ca; 7 Division of Neurology, Dalhousie University, Halifax, NS B3H4R2, Canada; david.larkspur@gmail.com; 8 Division of Movement Disorders, New York University Langone Medical Center, New York, NY 10016, USA; Steven.Frucht@nyulangone.org; 9 Department of Neurology, Icahn School of Medicine at Mount Sinai, New York, NY 10029, USA; david.simpson@mssm.edu

**Keywords:** botulinum toxin, tremor, upper-limb essential tremor, incobotulinumtoxinA, Xeomin, kinematics, clinical-decision support, treatment planning, TremorTek^®^

## Abstract

In this first, double-blind, randomized, placebo-controlled exploratory trial, we evaluate the efficacy and safety of incobotulinumtoxinA and feasibility of using kinematic tremor assessment to aid in the planning of muscle selection in a multicenter setting. Reproducibility of the planning technology to other clinical sites was explored. In this trial (NCT02207946), patients with upper-limb essential tremor (ET) were randomized 2:1 to a single treatment cycle of incobotulinumtoxinA or placebo. A tremor kinematic analytics investigational device was used to define a customized muscle set for injection, related to the pattern of the wrist, forearm, elbow, and shoulder tremor for each patient, and the incobotulinumtoxinA dose per muscle (total ≤ 200 U). Fahn–Tolosa–Marin (FTM) Part B motor performance score, Global Impression of Change Scale (GICS), and kinematic analysis-based efficacy evaluations were assessed. Thirty patients were randomized (incobotulinumtoxinA, *n* = 19; placebo, *n* = 11). FTM motor performance scores showed greater improvement with incobotulinumtoxinA versus placebo at Week 4 (*p* *=* 0.003) and Week 8 (*p* *=* 0.031). The physician-rated GICS score indicated improvement with incobotulinumtoxinA versus placebo at Week 4 (*p* < 0.05). IncobotulinumtoxinA also decreased accelerometric hand-tremor amplitude versus placebo from baseline to Week 4 (*p* *=* 0.004) and Week 8 (*p* < 0.001), with persistent tremor reduction up to 24 weeks post-injection. IncobotulinumtoxinA produced a slight and transient reduction of maximal grip strength versus placebo; two patients reported localized finger muscle weakness. Customized incobotulinumtoxinA injections decreased tremor severity and improved hand motor function in patients with upper-limb ET after a single injection cycle, with a favorable tolerability profile. The study showed that tremor kinematic analytics technology could be successfully scaled for use in other clinical sites.

## 1. Introduction

Essential tremor (ET), a common movement disorder affecting 4.6% of individuals ≥65 years of age [1], is characterized by uncontrollable trembling of the upper limbs [2]. Other areas, including the head, face, jaw, and vocal cords, can also be affected [2,3,4]. ET can significantly impair activities of daily living (ADL) [3,5].

Treatment is based on severity and impact on the quality of life [5]. Propranolol and primidone, the most commonly used therapies, reduce tremor amplitude by ~50% [2]. However, approximately 30–70% of patients fail to respond, and side effects are relatively common [2,6].

Two placebo-controlled studies evaluated botulinum neurotoxin type A (BoNT-A; onabotulinumtoxinA) in patients with hand ET [7,8]. The first study randomized 133 patients to onabotulinumtoxinA 50 U, 100 U or placebo injected into wrist muscles (flexor carpi radialis and ulnaris, extensor carpi radialis and ulnaris) for 16 weeks [7]. The authors reported a significant reduction in postural tremor with onabotulinumtoxinA versus placebo at 6, 12 and 16 weeks and in kinetic tremor at 6 weeks using a tremor severity rating scale; however, grip strength significantly reduced with onabotulinumtoxinA versus placebo, hand weakness was reported as an adverse event by half of the patients overall, and only minimal changes in motor function and functional disability occurred. The second study randomized 25 patients to onabotulinumtoxinA 50 U or placebo into the same wrist muscles as the first study for 16 weeks [8]. The authors reported a significant reduction in postural tremor by Week 4, which was maintained for the duration of the study; similar to the first study, finger weakness was reported by up to half of patients receiving onabotulinumtoxinA versus none receiving placebo, and functional rating scales did not significantly improve with onabotulinumtoxinA. Due to concerns of increased risk of hand or finger muscle weakness [7,8], BoNT-A has not been widely adopted for ET. However, there is growing evidence that a more customized selection of muscles and doses reduces muscle weakness and improves activities of daily living and quality of life of patients with ET [9,10,11,12].

Characterization of movement is important for selecting BoNT-A dose and the muscles to be injected, and is difficult to define in the upper limb due to complex joint biomechanics [13]. Kinematic analysis to record the dynamics of movement is well established for analysis of upper-limb movement [14,15] and has demonstrated benefits in improving the outcome of BoNT-A treatment for upper-limb Parkinson’s disease tremor [13,16]. Two open-label single-site studies of incobotulinumtoxinA were conducted in patients with ET [12,17,18]. The first study enrolled 31 patients to receive three incobotulinumtoxinA injections over 30 weeks and captured upper limb kinematics using goniometers and a torsiometer at the forearm, wrist, elbow and shoulder joints; the computerized tremor analysis was used to aid and plan a customized dosing algorithm [12]. Tremor amplitude was significantly reduced as early as 6 weeks and maintained throughout the treatment course, with only mild but not bothersome hand weakness and a reduction in grip strength that did not substantially impact hand function [12]. The second study enrolled 24 patients to receive up to six incobotulinumtoxinA treatments for up to 96 weeks [17,18]. Motion sensor devices on the forearm, wrist, elbow and shoulder captured tremor severity, and were used to select the most appropriate muscles for injection. A significant decline in tremor severity was reported as early as 6 weeks and maintained throughout the study course; perceived muscle weakness was not long-lasting, and the vast majority of patients experienced a functional benefit from treatment [17,18]. The present study investigated whether this promising approach was reproducible under placebo-controlled conditions in a multicenter setting, with the objective of assessing the efficacy and safety of a single, kinematic planned intramuscular injection of incobotulinumtoxinA, compared with placebo, in moderate-to-marked ET of the upper limb.

## 2. Results

### 2.1. Study Population

**Figure 1:**
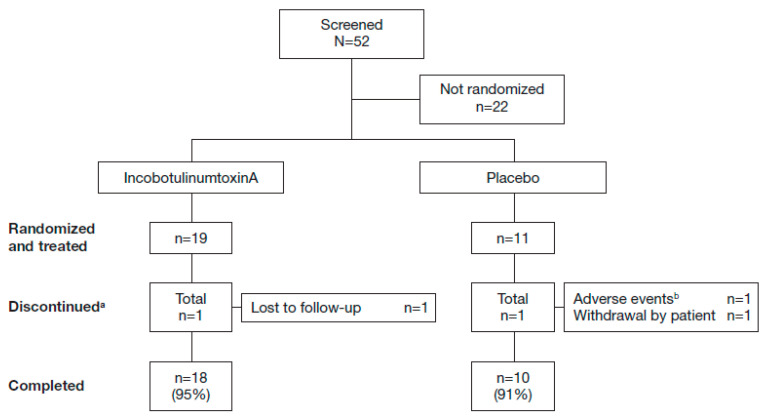
Patient disposition. ^a^ Multiple entries possible; ^b^ Serious adverse event of chest discomfort, not related to study medication.

A total of 30 patients were randomized to treatment, 19 received incobotulinumtoxinA, and 11 received placebo. Of these, one patient in the incobotulinumtoxinA group was lost to follow-up, and one patient in the placebo group withdrew from the study due to a treatment-emergent adverse event (TEAE) considered unrelated to treatment (Figure 1).

Patient demographics were broadly similar in the two groups at baseline. (Table 1). In total, 15/30 (50.0%) patients were female, the overall mean age was 68.1 years, and 28/30 (93.3%) patients were documented as having received concomitant medications. Beta-blocking agents and anti-epileptics, primarily prescribed to treat ET and kept at a stable dose from at least 4 weeks before screening until the study end, were documented for 52.6% and 26.3% of patients, respectively, in the incobotulinumtoxinA group and for 36.4% of patients each in the placebo group. The majority of patients (28/30) had a duration of ET > 3 years, with only two experiencing ET for ≤3 years: one for 1 year, and one for 2 years. No tremors had dystonic characteristics.

Patients received a mean (SD) dose of incobotulinumtoxinA 116.3 (53.0) U, and an equivalent volume was injected in the placebo group. Details of muscles injected and the mean incobotulinumtoxinA doses per muscle are shown in Table 2. Overall, patients received injections in a mean (SD) of 11.7 (3.8) and 12.2 (3.7) sites in the incobotulinumtoxinA and placebo groups, respectively. For most patients (15/19 [78.9%] in the incobotulinumtoxinA group and 8/11 [72.7%] in the placebo group), the actual dose was equal to the planned dose (based on kinematic tremor analysis) for all muscles. For two patients in each group the actual dose was not equal to the planned dose, and for the remaining patients no planned dose was documented. For the patients in the incobotulinumtoxinA group, a slight dose increase was documented in 1 patient (130 U instead of 128 U) due to the dose injected into the wrist muscle flexor carpi radialis (25 U) being above the recommended range of 5 to 20 U, while small variations occurred in the dosing of most muscles, and in the other patient, the injected total dose was reported as 110 U, identical to the planned dose, but the individual muscle doses showed a slightly different dose distribution. No sub-analysis was performed in patients who received the planned dose versus those who received a different dose due to the small numbers in the latter group.

### 2.2. FTM Tremor Rating and Motor Performance

**Figure 2:**
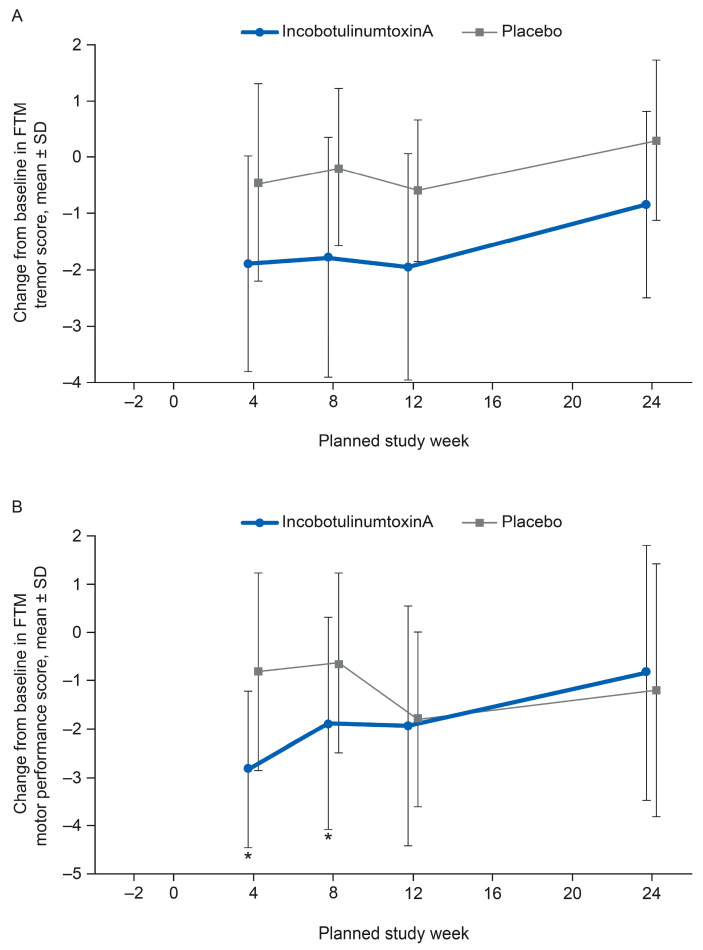
FTM tremor rating and motor performance. (**A**) FTM tremor score (injected limb), mean changes from baseline (±SD) (FAS, observed cases), and (**B**) FTM motor performance score (injected limb), mean changes from baseline (±SD) (FAS, observed cases). * *p* < 0.05 for incobotulinumtoxinA versus placebo (ANCOVA). Lower scores indicate better results. ANCOVA, analysis of covariance; FAS, full analysis set; FTM, Fahn–Tolosa–Marin.

Throughout the study, greater improvements in Fahn–Tolosa–Marin (FTM) tremor and Part B motor performance scores [19] were observed with incobotulinumtoxinA treatment versus placebo. There was a somewhat greater improvement from baseline in FTM tremor score in the incobotulinumtoxinA group versus placebo at Weeks 4 and 8, but the difference between the treatment groups was statistically significant favoring incobotulinumtoxinA at Week 8 only, when looking at the 95% confidence intervals based on the t-distribution [−3.1; −0.1], and not for Week 4 [−2.9; 0.0]. The sensitivity analysis of covariance (ANCOVA) did not show statistically significant differences at Week 4 (*p =* 0.159) or Week 8 (*p =* 0.087) (Figure 2A). A statistically significantly greater improvement from baseline in FTM Part B motor performance score was seen with incobotulinumtoxinA versus placebo at Weeks 4 and 8 (sensitivity ANCOVA; Week 4: least squares (LS) mean difference −2.3 [95% CI: −3.8, −0.9], *p =* 0.003; Week 8: LS mean difference −1.6 [95% CI: −3.0, −0.2], *p =* 0.031) (Figure 2B). Only two patients in each treatment group had their non-dominant hand treated, and a subgroup analysis (dominant hand only vs. non-dominant hand only) confirmed the results of the FTM Part B motor performance score analysis.

### 2.3. Global Impression of Change

The physician-rated global impression of change scale (GICS) scores indicated statistically significantly greater improvement in the incobotulinumtoxinA group versus placebo at Week 4 (95% CI: 0.2, 1.3; *p* < 0.05 based on t-distribution for the treatment; Figure S1), but there were no significant differences at any other timepoint. A somewhat greater improvement with incobotulinumtoxinA versus placebo was observed for patient-rated GICS at Week 4, but this was not statistically significant (95% CI: −0.2, 0.8; *p* > 0.05; Table S1).

### 2.4. Kinematic Tremor Analysis

**Figure 3:**
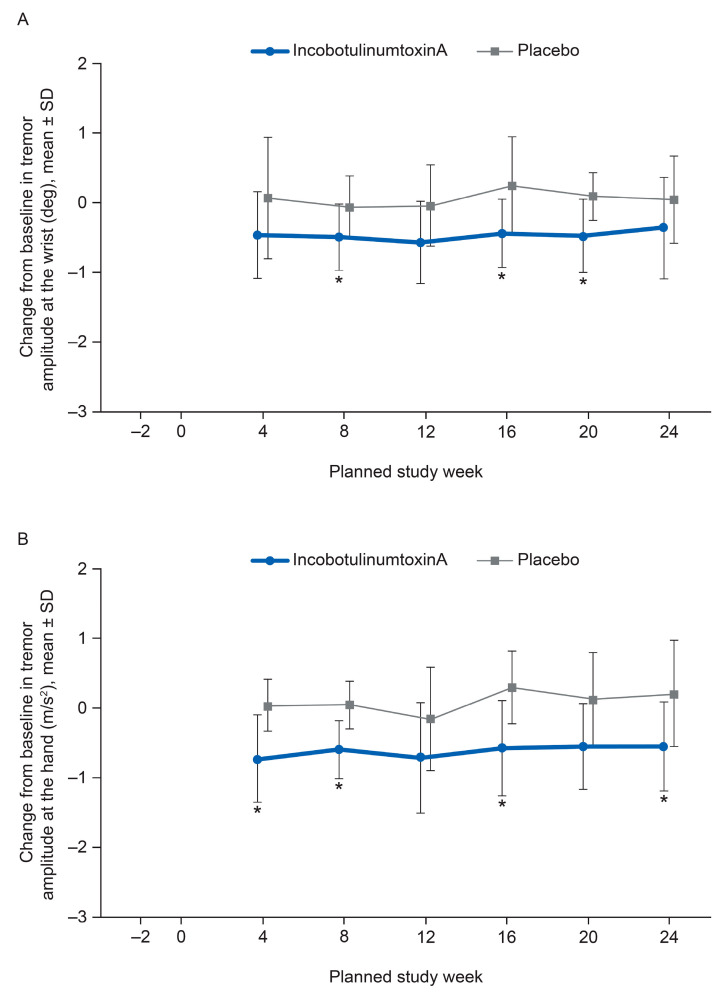
Kinematic tremor analysis. (**A**) Maximum angular tremor amplitude at the wrist (injected limb), mean change from baseline (FAS, observed cases, (deg)), and (**B**) maximum log-transformed accelerometric tremor amplitude at the hand (injected limb), mean change from baseline (±SD) (FAS, observed cases, (m/s^2^)). * *p* < 0.05 for incobotulinumtoxinA versus placebo (ANCOVA). Lower scores indicate better results. ANCOVA, analysis of covariance; deg, degrees of arc; FAS, full analysis set.

IncobotulinumtoxinA significantly decreased maximum log-transformed accelerometric hand-tremor amplitude from baseline versus placebo at Weeks 4 and 8 (sensitivity ANCOVA; Week 4: LS mean difference (incobotulinumtoxinA–placebo) −0.66 m/s^2^ (95% CI: −1.09, −0.23), *p =* 0.004; Week 8: LS mean difference −0.59 m/s^2^ (95% CI: −0.92, −0.27), *p* < 0.001) (Figure 3B). Kinematic outputs also showed persistent amelioration of tremor from baseline beyond Week 8 to Week 24 post-injection, with statistical significance versus placebo at Weeks 16 and 20 for maximum wrist angular tremor amplitude and Weeks 16 and 24 for maximum log-transformed accelerometric hand-tremor amplitude (Figure 3). Furthermore, incobotulinumtoxinA decreased maximum wrist angular tremor amplitude (root mean square (RMS)) from baseline versus placebo at Week 8 (sensitivity ANCOVA; Week 4: LS mean difference (incobotulinumtoxinA–placebo) −0.43 RMS degrees [95% CI: −1.02, 0.16], *p =* 0.144; Week 8: LS mean difference −0.41 RMS degrees [95% CI: −0.69, −0.14], *p =* 0.005) (Figure 3A).

### 2.5. Safety Outcomes

The incidence of TEAEs was generally similar between the incobotulinumtoxinA and placebo groups. TEAEs were reported by 9/19 (47.4%) and 6/11 (54.5%) patients in the incobotulinumtoxinA and placebo groups, respectively. TEAEs considered to be related to treatment occurred in 3/19 (15.8%) and 1/11 (9.1%) patients in the incobotulinumtoxinA and placebo groups, respectively (Table S2). In the incobotulinumtoxinA group, two patients experienced muscular weakness localized to the finger extensors, and one other patient had bruising and pain at the injection site. In the placebo group, one patient experienced contusion and asthenia. All TEAEs considered related to treatment were of mild intensity. In the incobotulinumtoxinA group, there were no serious TEAEs, or TEAEs leading to discontinuation. In the placebo group, 2/11 (18.2%) patients experienced serious TEAEs of influenza-like illness and chest discomfort; both considered unrelated to the study medication. The serious TEAE of chest discomfort led to study discontinuation. No patients died during this study.

Three patients in the incobotulinumtoxinA treatment group experienced four TEAEs of special interest: localized muscular weakness (*n* = 2), dry mouth (*n* = 1), and dysphonia (*n* = 1). One patient experienced both localized muscular weakness and dry mouth. In the placebo group, one patient experienced dysphagia. All TEAEs of special interest were of mild intensity, and only the two events of localized muscular weakness (excluding dry mouth) were assessed as related to treatment by the investigator. In both cases of muscular weakness, a general trend toward a decrease in maximal grip strength over the first 8 weeks was observed.

**Figure 4:**
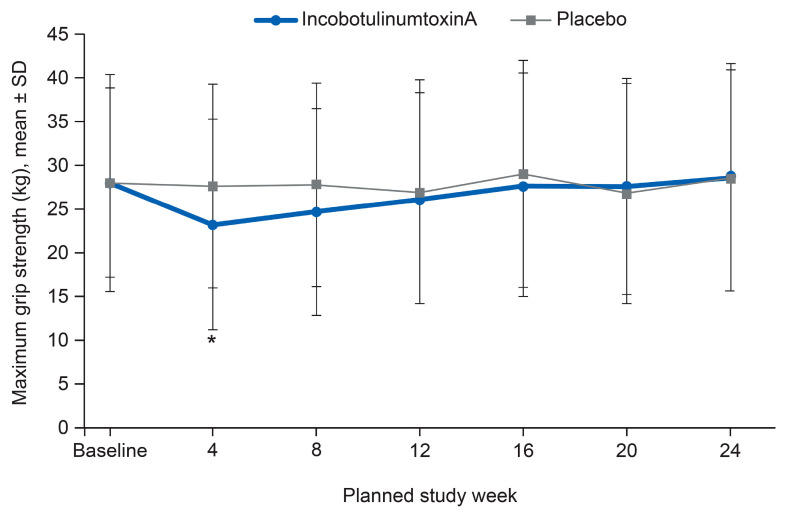
Maximum grip strength using hand-held dynamometers (injected limb) (safety evaluation set). * *p* < 0.05, 95% CI based on t-distribution for the difference between treatment groups.

The average maximum grip strength in the hand of the injected arm was reduced after incobotulinumtoxinA treatment. At Week 4 post-injection, the mean (SD) maximum grip strength was 23.32 (12.04) kg and 27.64 (11.58) kg in the incobotulinumtoxinA and placebo groups, respectively, and the difference between the treatment groups was statistically significant (*p* < 0.05, 95% CI: −8.96, −1.56; based on t-distribution for the difference between treatment groups). However, grip strength subsequently returned to baseline levels, and there was no significant difference from baseline in either group by Week 24 (Figure 4).

Mean muscle strength in the injected limb (MRC MMT), was also significantly reduced at Week 4 versus baseline after incobotulinumtoxinA treatment (Table S3). However, there were no significant changes from baseline in self-perceived weakness of the treated arm or hand in patients who received incobotulinumtoxinA or placebo (Table S4).

## 3. Discussion

In this randomized, double-blind, placebo-controlled clinical trial in patients with moderate-to-marked ET of the upper limb, all efficacy variables consistently favored the incobotulinumtoxinA group after a single treatment cycle, until at least Week 12. Kinematic planned incobotulinumtoxinA treatment statistically significantly and persistently decreased tremor severity for up to 24 weeks post-injection, with greater improvement in physician-rated GICS versus placebo 4 weeks post-injection. IncobotulinumtoxinA-treated patients experienced a slight and transient reduction in maximal grip strength versus placebo, but no significant effect on self-perceived muscle weakness. IncobotulinumtoxinA conferred a statistically significant improvement in FTM part B score for motor function at Weeks 4 and 8 versus placebo, suggesting that the observed transient reduction in maximal grip strength did not inhibit the patients’ ability to perform coordinated motor tasks. Notably, although greater improvements in FTM tremor score were observed with incobotulinumtoxinA versus placebo at Weeks 4 and 8, these were not statistically significant at Week 4. The contrast between FTM tremor and Part B motor performance scores may have been due to the tremor score being visually assessed and subjective, which causes crude variabilities, while the motor performance score is a more reliable measure of improvement. Results from this multicenter study highlight the reproducibility of this technique and are consistent with those of a recent open-label study, in which kinematic planned incobotulinumtoxinA injections for ET resulted in a reduction in tremor amplitude and significant improvement in arm function, despite mild, yet non-bothersome, weakness in the treated muscles [17,18].

The effective treatment of ET remains an unmet clinical need [5,20]. Although propranolol and primidone have shown efficacy in ET, many patients fail to respond, and side effects are common [6]. Consistent with this, the inclusion criteria for the current study required that patients had a moderate-to-marked tremor and any patients taking concomitant anti-tremor medication received a stable dose, from at least 4 weeks prior to the screening visit until the study end. The high proportion of patients (93.3%) on concomitant beta-blocking agents and anti-epileptics while still meeting the inclusion criteria of moderate-to-marked tremor, indicates that the majority had an inadequate therapeutic effect with these therapies prior to the study.

Based on recent evidence, it has been postulated that the symptoms of ET may be part of degenerative brain pathophysiology characterized by structural changes to the cerebellum, particularly the Purkinje cell population [21]. Side effects of currently available oral therapies may result in a reduction of cerebral performance, including confusion and cognitive difficulties [2,5]. Therefore, a localized treatment, such as BoNT, may provide an alternative to a systemic oral approach, or too invasive, complication-prone treatment such as deep brain stimulation.

Two previous studies followed a standard non-personalized protocol for the injection of wrist muscles with onabotulinumtoxinA. One study injected onabotulinumtoxinA 15 U or 30 U into each of the flexor carpi radialis and ulnaris and 10 U or 20 U into each of the extensor carpi radialis and ulnaris. Significant improvements from baseline in postural and kinetic tremor at 6 weeks were reported, with significant reduction in grip strength [7]. The second study injected onabotulinumtoxinA 15 U into each flexor carpi radialis and flexor carpi ulnaris and 10 U into each extensor carpi radialis and extensor carpi ulnaris. The authors reported improvements in postural tremor and treatment response ratings, but no significant improvement in functional score with BoNT-A versus placebo [8]. In both studies, the extent of the improvement was limited by the development of functional impairment caused by disproportionate wrist and hand weakness [7,8]. In one study, depending on the administered BoNT-A dosage, up to 70% of patients experienced weakness in the fingers and/or wrist, as measured by maximum grip strength and MRC clinical ratings, which impacted ADL in some patients [7].

In light of this evidence, the American Academy of Neurology recommended that the benefits of BoNT-A should be considered in the context of the “common adverse effect of muscle weakness” and that BoNT should be considered as a treatment option for ET of the hand in those patients who fail to respond to oral therapy [22]. Results of a more recent single injection placebo-controlled study suggest that personalized injections with a broader spectrum of injected muscles improves hand weakness [9]. However, selection of the tremor-causing muscles remains challenging. While Mittal et al. used electromyography to identify rhythmic burst potentials, the kinematic analyses for each plane of motion used in this study appears to provide a standardized and reproducible approach with additional help to aid the injector in the customization of dose selection for individual muscles [12]. Furthermore, the customized protocol used in this study, combined with the significantly reduced MRC MMT scores at Week 4, indicate that this protocol may overcome the concerns seen in previous trials using a standard protocol.

In the current study, customized dosing patterns on kinematic tremor analysis was well tolerated: only two patients (10.5%) in the incobotulinumtoxinA group experienced muscular weakness localized to the fingers of the injected limb. No patient discontinued the study due to muscle weakness.

Furthermore, although the effect of muscle weakness on ADL was not assessed in this study, mean MRC MMT scores in both the incobotulinumtoxinA and placebo groups ranged between 24.1 and 25.0 at all post-injection visits. As a maximum score of 25 on the MRC MMT scale indicates normal muscle strength, these scores demonstrate overall good muscle strength, which one may assume had little or no impact on ADL. The results suggest that customized incobotulinumtoxinA injections can be a promising treatment for ET with a lower rate of side effects than shown in previous fixed-dose studies.

This study demonstrated the efficacy and tolerability of incobotulinumtoxinA for the treatment of ET and the feasibility of using kinematic analysis at multiple sites. While the study was exploratory in nature, lacked a primary endpoint and was not powered to demonstrate the superiority of incobotulinumtoxinA versus placebo, statistically significant improvements in efficacy were shown with incobotulinumtoxinA; these findings would need to be confirmed in larger studies. A further limitation was the single treatment cycle. Measurements over additional treatment cycles would allow better estimation of long-term safety and may demonstrate enhanced treatment efficacy, as shown previously [17]. Using such an approach in a recent open-label pilot study, a continuous trend of further improvements in FTM scales was noted up to 96 weeks when follow-up treatments were further optimized with kinematic based outcome measures [18].

## 4. Conclusions

This double-blind, exploratory, placebo-controlled study adds further evidence that customized incobotulinumtoxinA dosing is well tolerated and effective for the treatment of ET, with treatment effects lasting at least up to 24 weeks. The study showed that tremor kinematic analytics (TremorTek^®^) technology could be successfully adopted in other clinical sites. A confirmatory long-term study is planned to investigate the efficacy and tolerability of customized incobotulinumtoxinA injections for the treatment of ET in a larger patient population, and to further elucidate the usefulness of kinematic planning technology.

## 5. Materials and Methods

### 5.1. Study Design and Patients

This trial was a prospective, randomized, double-blind, placebo-controlled, parallel-group, multicenter, exploratory study in patients with upper-limb ET (NCT02207946). Patients were enrolled from December 2014 to April 2016 by six investigators in Canada and the United States (US) (at Medicine Professional Corporation, Toronto, ON, Canada; Movement Disorders Clinic, Toronto Western Hospital, Toronto, ON, Canada; Movement Disorder Clinic, Winnipeg, MB, Canada; David King, Inc., Halifax, NS, Canada; Mount Sinai Medical Center, New York, NY, USA).

Eligible patients were adults with a first onset of ET ≥6 months before screening and with stability of symptoms over 4 weeks; whose diagnosis of “definite ET” was otherwise in accordance with the Tremor Investigation Group criteria applicable at the time [23] (bilateral postural tremor with/without kinetic tremor, involving hands and forearms, that was visible and persistent); moderate-to-marked upper-limb postural and/or kinetic tremor at wrist level (FTM Part C, items 17–23) in the limb to be treated, with a rating of ≥2 in at least two categories; visible tremor at wrist level in at least one of the four positions/tasks used in kinematic assessment; tremor deemed by the investigator to require treatment with a total dose of 30–200 U incobotulinumtoxinA for up to 3 joints (wrist joint mandatory; shoulder and elbow joints optional); and receiving concomitant anti-tremor medication, if any, at a stable dose (≥4 weeks before screening and until the study end). Principal exclusion criteria are listed in the supplementary materials.

### 5.2. Treatment

Patients were randomized 2:1 to receive, in a single treatment cycle, unilateral, intramuscular injections of incobotulinumtoxinA (total dose 30–200 U), or an equivalent volume of placebo, into muscles of the wrist (mandatory) and, optionally, into the elbow and/or shoulder, of the limb that (in the patient’s opinion) had the greatest impact on their ADL (the doses received are shown in Table 2). Injections were guided using ultrasound, electromyography, and/or electrical nerve stimulation as determined by the investigator. Injections were conducted by experienced investigators familiar with the Pictorial Atlas of Botulinum Toxin Injection: Dosage, Localization, Application by Jost [24].

The treatment planning for muscles to be treated was based on kinematic tremor analysis using the tremor kinematic analytics investigational device, and followed kinematic tremor procedures as previously described [17,18] (this technology is pending regulatory approval, and is available to clinical investigators for research purposes from the corresponding author). The customized treatment pattern for total dose and dosing regimen was based on kinematic tremor analysis; however, this clinical decision algorithm could be modified at the investigator’s discretion. Patients were monitored regularly over 24 weeks, including telephone contact 1 week post-injection and clinic visits every 4 weeks until the end-of-study visit at Week 24.

### 5.3. Efficacy Assessments

No primary efficacy variables were defined in this exploratory study. Secondary efficacy variables included change from baseline to Week 4 in: maximum angular tremor amplitude of the wrist of the injected limb; maximum log-transformed accelerometric tremor amplitude at hand level of the injected limb; FTM tremor score in the injected limb [19]; FTM motor performance score [19]; and physicians’ and patients’ GICS score at Week 4. Changes from baseline to later time points from Weeks 8–24 were assessed every 4 weeks as further efficacy variables.

### 5.4. Kinematic Tremor Analysis

The maximum log-transformed accelerometric tremor amplitude at the hand and the RMS angular tremor amplitude at the shoulder, elbow, forearm, and wrist of the injected limb were measured by kinematic tremor analysis at the screening and baseline visits, and every 4 weeks from Week 4 to Week 24 post-injection. The analysis was conducted as described previously [16,17].

#### 5.4.1. FTM Tremor Rating Scale

Tremor severity and functional effects were assessed by the investigator using the FTM tremor rating scale [19] at screening and baseline visits, and Weeks 4, 8, 12, and 24 post-injection. The change from baseline in FTM tremor score for the injected limb was assessed using FTM Part A items 5 or 6 (right or left upper limb, respectively) for three functions (at rest, with posture holding, with action and intention). The scale ranged from 0 (normal) to 4 (severe, amplitude > 4 cm) for each item and from 0 (normal) to 12 (severe) for the sum score of all three functions.

The change from baseline in FTM motor performance score was calculated from the sum of FTM Part B items 11 (handwriting for the dominant hand, irrespective of treatment) and 12–15 (drawing a large/small spiral, drawing a line and pouring for the treated limb only). The scale ranged from 0 (normal) to 4 (severe) for each item and from 0 (normal) to 20 (severe) for the sum of all five motor tasks.

#### 5.4.2. Global Impression of Change

The physicians’ and patients’ GIC were assessed using the GICS, a 7-point Likert scale [25] from −3 (very much worse) to +3 (very much improved) every 4 weeks from Week 4 to Week 24 post-injection.

### 5.5. Safety Assessments

#### 5.5.1. Adverse Events

Adverse events were monitored, and predefined adverse events of special interest thought to be a possible indication of toxin spread, based on Medical Dictionary for Regulatory Activities-preferred terms (version 19.0), were monitored by targeted questioning. TEAEs, including those of special interest, were analyzed. These were defined as adverse events with onset or worsening at/after date and time of the first administration of incobotulinumtoxinA.

#### 5.5.2. Other Safety Assessments

Maximum grip strength (kg) was measured for both hands using a hand-held dynamometer. Details of additional safety assessments (MRC MMT and self-perceived weakness) are presented in the supplementary materials.

### 5.6. Statistical Analysis

No formal sample size calculation was performed, and all analyses were exploratory. Two-sided 95% CIs (based on the t-distribution) were calculated for the change from baseline and for treatment differences for efficacy variables. The treatment groups were compared using sensitivity ANCOVA, mixed model repeated measures (MMRM), and the Wilcoxon rank-sum test, using observed cases. Descriptive *p*-values from ANCOVA, MMRM, and the Wilcoxon rank-sum test were provided as appropriate.

Efficacy analyses were based on the full analysis set (all treated patients with a post-baseline value for any efficacy variable) and on the per-protocol population (patients in the full analysis set, for whom no major protocol violations were recorded) for the sensitivity analysis. Safety data were based on the safety evaluation set (all patients who received study medication) and summarized descriptively. Statistical analyses were performed using SAS^®^ version 9.2 (SAS Institute, Cary, NC, USA).

### 5.7. Standard Protocol Approvals, Registrations, and Patient Consents

The study was conducted in accordance with the International Conference on Harmonization Guidelines, applicable regulations, and the principles of the Declaration of Helsinki. The study protocol was reviewed by an independent ethics committee or institutional review board at each study site. The study was approved by the: Western Institutional Review Board, Puyallup, WA, Canada; University Health Network Research Ethics Board, Toronto, Canada; University of Manitoba Bannatyne Campus Research Ethics Board, Manitoba, Canada; and BRANT IRB Accreditation Consulting Services, Lake Success, New York, NY, USA (further details in Table S5). All patients provided written informed consent.

## Figures and Tables

**Table 1:** Patient demographics (safety evaluation set).

Characteristic	IncobotulinumtoxinA (*n* = 19)	Placebo (*n* = 11)	Total (*n* = 30)
**Sex, *n* (%)**			
Female	10 (52.6)	5 (45.5)	15 (50.0)
Male	9 (47.4)	6 (54.5)	15 (50.0)
**Age, years**			
Mean (SD)	68.1 (10.6)	68.2 (10.2)	68.1 (10.3)
Min, max	41, 88	49, 85	41, 88
**Ethnic origin, *n* (%)**			
White	17 (89.5)	11 (100.0)	28 (93.3)
Black or African American	1 (5.3)	0 (0.0)	1 (3.3)
Other	1 (5.3)	0 (0.0)	1 (3.3)
**Time since onset of ET, years; mean (SD)**	24.7 (19.6)	35.3 (26.4)	28.1 (22.4)
**Concomitant medication for ET, *n* (%) ^a^**			
Beta-blockers ^b^	10 (52.6)	4 (36.4)	14 (46.7)
Anti-epileptics ^c^	5 (26.3)	4 (36.4)	9 (30.0)

^a^ Concomitant anti-tremor medication on a stable dose from at least 4 weeks before screening and until the end of the study. Also taken in the incobotulinumtoxinA group were agents acting on the renin-angiotensin system (47.4%), lipid-modifying agents (36.8%), and antithrombotic agents, anti-inflammatory and antirheumatic products, and drugs used in diabetes (26.3% each). ^b^ Beta-blockers included propranolol, metoprolol, propranolol hydrochloride, and carvedilol. ^c^ Anti-epileptics included primidone, gabapentin, lamotrigine, topiramate, and phenytoin. ET, essential tremor.

**Table 2:** Frequency of muscles injected in the placebo and incobotulinumtoxinA groups and mean injected dose of incobotulinumtoxinA (safety evaluation set).

Clinical Pattern	IncobotulinumtoxinA (N = 19)		Placebo (N = 11)
Muscle	*n*	Dose, U; Mean (SD)	*n*
**Total**	19	116.3 (53.0)	11
**Wrist (mandatory treatment) ^a,b^**			
Total	19	47.4 (18.1)	11
Extensor carpi radialis	14	9.6 (6.0)	10
Extensor carpi ulnaris	11	8.6 (3.9)	8
Flexor carpi radialis	16	10.6 (5.1)	11
Flexor carpi ulnaris	16	7.2 (2.6)	7
Pronator quadratus	13	8.1 (4.8)	8
Pronator teres	14	8.9 (4.5)	8
Supinator	15	10.3 (6.4)	7
**Shoulder ^c^**			
Total	14	45.0 (7.6)	9
Deltoid	5	9.0 (2.2)	1
Latissimus dorsi	12	11.3 (3.8)	8
Pectoralis major	14	21.1 (4.5)	9
Supraspinatus	14	11.1 (4.0)	8
Teres major	0	0.0 (0.0)	1
**Elbow ^d^**			
Total	16	42.5 (13.9)	10
Biceps brachii	2	30.0 (0.0)	1
Brachialis	14	20.0 (6.5)	9
Triceps brachii	16	21.3 (7.0)	10

Total customized dose, 30–200 U per patient. Maximum dose per injection site, 25 U. For each patient, the dose per muscle was determined using kinematic analysis, which helped to prioritize muscles requiring treatment. Patients in the placebo group received an equivalent volume of placebo based on their customized dose calculation for treatment. ^a^ Dose range, 5–20 U per muscle (1 injection site); maximum total dose 80 U. ^b^ The wrist required at least 30 U of incobotulinumtoxinA, with a maximum dose of 20 U per muscle. Thus, at least two muscles of the wrist were treated. Note that pronator quadratus, pronator teres and supinator cause tremor motion around the forearm. ^c^ Dose range, 10–40 U per muscle (1–3 injection sites); maximum total dose 60 U. ^d^ Dose range, 15–40 U per muscle (2 injection sites); maximum total dose 60 U. N, number of patients; *n*, number of observations.

## Supplementary Materials

The following are available online at https://www.mdpi.com/2072-6651/12/12/807/s1, Supplementary methods; Table S1: Physician’s and patient’s mean global impression of change scale scores at Weeks 4 and 8 (full analysis set); Table S2: Overall summary of treatment-emergent adverse events (safety evaluation set); Table S3: Medical Research Council manual muscle testing total scores and change from baseline (safety evaluation set); Table S4: Change from baseline in self-perceived weakness of the arm and hand (safety evaluation set); Table S5: Details of study sites and EC, CEC, LEC and IRB; Figure S1: (A) Physician’s and (B) patient’s GICS at Weeks 4, 8, and 12 (full analysis set).

## Data Sharing Statement

Information on this study is available to researchers and can be found in the clinicaltrials.gov record (NCT02207946). All relevant data are within this paper and its online supplementary material files.

## Abbreviations

ADL	Activities of daily living
ANCOVA	Analysis of covariance
BoNT-A	Botulinum neurotoxin type A
CI	Confidence interval
ET	Essential tremor
FAS	Full analysis set
FTM	Fahn–Tolosa–Marin
GICS	Global Impression of Change Scale
IRB	Institutional review board
LS	Least squares
MMRM	Mixed model repeated measures
MRC	Medical Research Council
MRC MMT	Medical Research Council manual muscle testing
RMS	Root mean square
SD	Standard deviation
TEAE	Treatment-emergent adverse event
U	Unit
