# Supplementary material for: Tolerability and Efficacy of Customized IncobotulinumtoxinA Injections for Essential Tremor: A Randomized, Double-Blind, Placebo-Controlled Study
Source: Toxins (Basel). 2020 Dec 20;12(12):807. doi: 10.3390/toxins12120807 (PMC7766785; doi:10.3390/toxins12120807)
Supplement: Supplementary file 1 [file toxins-12-00807-s001.pdf]

Supplementary Materials: Tolerability and Efficacy of Customized IncobotulinumtoxinA Injections for Essential Tremor: A Randomized, Double-blind, Placebo-Controlled Study

Mandar Jog, Jack Lee, Astrid Scheschonka, Robert Chen, Farooq Ismail, Chris Boulias, Douglas Hobson, David King, Michael Althaus, Olivier Simon, Hanna Dersch, Steven Frucht, David M. Simpson and on behalf of the Essential Tremor Study Team

Supplementary methods

1. Exclusion Criteria

Major exclusion criteria included: any abnormal neurologic signs other than tremor and Froment's maneuver (rigidity in the wrist associated with the movement of the contralateral limb [1,2]); current exposure to lithium, valproic acid, amiodarone, neuroleptics or any tremorogenic/potentially tremorogenic drugs that may interfere with evaluation of the study drug; significant trauma to the central nervous system or the nerves of the target limb during the 3 months preceding onset of tremor; prior surgery to treat tremor; historic/clinical evidence of psychogenic origins of tremor; life habits considered prejudicial to study participation (e.g. smoking, alcohol or substance abuse); treatment (<16 weeks prior to the study) with any botulinum toxin product for any reason; planned surgery or other specified relevant treatments and/or concomitant disorders, which in the opinion of the investigator, could affect the outcome of the study treatment.

2. Other Safety Assessments

2.1. Medical Research Council Manual Muscle Testing (MRC MMT)

At baseline and 4, 8, 12, 16, 20, and 24 weeks post-injection, the muscle strength of the injected limb was measured using the MRC MMT scale for muscle strength of all individual fingers of the treated hand at the level of the proximal interphalangeal joints. The patient's muscle strength at each individual joint was rated from 0 (no palpable or observable muscle contraction) to 5 (holds test position against maximal resistance: muscle contracts normally against full resistance); range 0–25 for the total score from the sum of five finger scores.

2.2. Self-Perceived Weakness

On the day of assessment, patients were asked to assess the weakness of their treated arm/hand on a 10-point Likert scale ranging from 0 (no perceived weakness) to 10 (worst imaginable weakness). The scores for the arm and the hand were assessed separately.

Supplementary Results

Table S1. Physician's and patient's mean global impression of change scale scores at weeks 4 and 8 (full analysis set).

	IncobotulinumtoxinA (*n* = 18)	Placebo (*n* = 11)
**Physician's GICS**		
Week 4, mean (SD)	0.8 (0.7)	0.1 (0.7)
95% CI from t-test	0.2, 1.3*	
Week 8, mean (SD)	0.7 (0.9)	0.1 (0.8)
95% CI from t-test	–0.1, 1.3	
**Patient's GICS**		
Week 4, mean (SD)	0.6 (0.6)	0.3 (0.6)
95% CI from t-test	–0.2, 0.8	
Week 8, mean (SD)	0.7 (0.8)	0.3 (0.8)
95% CI from t-test	–0.3, 1.0	

*P < 0.05, 95% CI based on t-distribution for the difference between treatment groups. GICS from –3 (very much worse) to +3 (very much improved); higher values indicate better results. GICS, global impression of change scale.

Table S2. Overall summary of treatment-emergent adverse events (safety evaluation set).

	IncobotulinumtoxinA (*n* = 19)	Placebo (*n* = 11)
**Number (%) of patients with any:**		
TEAE (at least 1)	9 (47.4)	6 (54.5)
Upper respiratory tract infection	2 (10.5)	2 (18.2)
Muscular weakness	2 (10.5)	0
Cystitis	1 (5.3)	0
Eyelid infection	1 (5.3)	0
Genital infection	1 (5.3)	0
Nail infection	1 (5.3)	0
Sinusitis	1 (5.3)	0
Tooth infection	1 (5.3)	0
Appendicitis	0	1 (9.1)
Back pain	1 (5.3)	0
Exostosis	1 (5.3)	0
Arthritis	0	1 (9.1)
Muscle spasms	0	1 (9.1)
Osteoarthritis	0	1 (9.1)
Ankle fracture	1 (5.3)	0
Head injury	1 (5.3)	0
Joint injury	1 (5.3)	0
Contusion	0	1 (9.1)
Diarrhea	1 (5.3)	0
Dry mouth	1 (5.3)	0
Dysphagia	0	1 (9.1)
Glaucoma	1 (5.3)	0
Injection site bruising	1 (5.3)	0
Injection site pain	1 (5.3)	0
Asthenia	0	1 (9.1)

Chest discomfort	0	1 (9.1)
Influenza-like illness	0	1 (9.1)
Blood cholesterol increased	1 (5.3)	0
Diabetes mellitus	1 (5.3)	0
Dysphonia	1 (5.3)	0
Hypoesthesia	0	1 (9.1)
Paresthesia	0	1 (9.1)
Breast mass	0	1 (9.1)
Related TEAE	3 (15.8)	1 (9.1)
TEAE of special interest	3 (15.8)	1 (9.1)
Related TEAE of special interest	2 (10.5)	0 (0.0)
Serious TEAE	0 (0.0)	2 (18.2)
Related serious TEAE	0 (0.0)	0 (0.0)
TEAE leading to discontinuation	0 (0.0)	1 (9.1)
Related TEAE leading to discontinuation	0 (0.0)	0 (0.0)
Fatal TEAE	0 (0.0)	0 (0.0)
Related fatal TEAE	0 (0.0)	0 (0.0)

TEAE, treatment-emergent adverse event.

Table S3. Medical Research Council manual muscle testing total scores and change from baseline (safety evaluation set).

Visit	IncobotulinumtoxinA (*n* = 19)				Placebo (*n* = 11)				95% CI[a], IncobotulinumtoxinA – placebo
	n	Total score, mean (SD)	Change from baseline, mean (SD)	95% CI[a]	*n*	Total score, mean (SD)	Change from baseline, mean (SD)	95% CI[a]	
Wk 4	18	24.1 (1.4)	–0.7 (1.4)	–1.4, 0.0	11	25.0 (0.0)	0.1 (0.3)	–0.1, 0.3	–1.6, –0.1*
Wk 8	18	24.4 (1.6)	–0.4 (1.7)	–1.3, 0.4	11	24.6 (1.2)	–0.3 (1.3)	–1.1, 0.6	–1.4, 1.1
Wk 12	18	24.6 (1.2)	–0.2 (1.4)	–0.9, 0.5	10	25.0 (0.0)	0.1 (0.3)	–0.1, 0.3	–1.0, 0.4
Wk 16	18	24.7 (1.0)	–0.2 (1.2)	–0.7, 0.4	10	25.0 (0.0)	0.1 (0.3)	–0.1, 0.3	–0.9, 0.3
Wk 20	18	24.8 (0.5)	0.0 (0.7)	–0.3, 0.3	10	25.0 (0.0)	0.1 (0.3)	–0.1, 0.3	–0.5, 0.3
Wk 24	18	25.0 (0.0)	0.2 (0.4)	0.0, 0.4	10	25.0 (0.0)	0.1 (0.3)	–0.1, 0.3	–0.2, 0.4

* Significant difference as 95% CI does not include 0 ($p \leq 0.05$). [a] 95% CI based on t-distribution for the difference from baseline for each treatment group and between treatment groups. n, number of observations; Wk, week..

Table 4. Change from baseline in self-perceived weakness of the arm and hand (safety evaluation set).

Visit	IncobotulinumtoxinA (n = 19)			Placebo (n = 11)			95% CI[a], IncobotulinumtoxinA – placebo
	n	Change from baseline, mean (SD)	95% CI[a]	n	Change from baseline, mean (SD)	95% CI[a]	
**Self-perceived arm weakness**							
Wk 4	18	–0.61 (3.35)	–2.27, 1.05	11	–0.18 (1.83)	–1.41, 1.05	–2.69, 1.83
Wk 8	18	–0.11 (4.42)	–2.31, 2.09	11	–0.36 (0.92)	–0.98, 0.26	–2.00, 2.51
Wk 12	18	–0.56 (3.60)	–2.35, 1.24	10	0.40 (0.84)	–0.20, 1.00	–2.81, 0.90
Wk 16	18	–0.83 (3.26)	–2.45, 0.79	10	0.10 (0.74)	–0.43, 0.63	–2.61, 0.74
Wk 20	18	–0.94 (3.28)	–2.58, 0.69	10	0.20 (1.03)	–0.54, 0.94	–2.88, 0.60
Wk 24	18	–1.28 (3.21)	–2.88, 0.32	10	0.00 (0.00)	0.00, 0.00	–2.88, 0.32
**Self-perceived hand weakness**							
Wk 4	18	–0.50 (3.35)	–2.16, 1.16	11	0.00 (2.24)	–1.50, 1.50	–2.84, 1.84
Wk 8	18	–0.28 (4.34)	–2.43, 1.88	11	0.09 (1.58)	–0.97, 1.15	–2.70, 1.96
Wk 12	18	–0.44 (3.79)	–2.33, 1.44	10	0.30 (0.67)	–0.18, 0.78	–2.67, 1.18
Wk 16	18	–1.11 (3.39)	–2.80, 0.58	10	0.10 (0.74)	–0.43, 0.63	–2.95, 0.53
Wk 20	18	–1.50 (3.40)	–3.19, 0.19	10	0.20 (0.63)	–0.25, 0.65	–3.43, 0.03
Wk 24	18	–1.56 (3.52)	–3.31, 0.19	10	0.00 (0.00)	0.00, 0.00	–3.31, 0.19

[a] 95% CI based on t-distribution for the difference from baseline for each treatment group and between treatment groups. n, number of observations; Wk, week.

Table S5. Details of study sites and EC, CEC, LEC and IRB.

Country	Site Number	Site Name and Address	Investigator Name	EC/CEC/LEC/IRB Name and Address	Initial Approval Date
Canada	0010305/1	Medicine Professional Corporation<br>82 Buttonwood Ave., Ruddy Building, 3rd Floor, Suite 3-94<br>Toronto, Ontario, M6M 2J5, Canada	Dr Christos Boulias	Western Institutional Review Board<br>1019 39th Avenue SE Suite 120<br>Puyallup, WA 98374-2115, Canada	August 4, 2014
Canada	0010078	University Health Network, Toronto Western Hospital<br>Department of Neurology – Movement Disorders<br>399 Bathurst Street MCL 7-402<br>Toronto, Ontario, M5T 2H7, Canada	Dr Robert Chen	University Health Network<br>Research Ethics Board<br>10th Floor, Room 1056<br>700 University Avenue<br>Toronto, Ontario, M5G 1Z5, Canada	January 7, 2015
Canada	0010317	Movement Disorder Clinic<br>200 Woodlawn Street<br>Winnipeg, Manitoba, R3J 2H7, Canada	Dr Doug Hobson	University of Manitoba<br>Bannatyne Campus<br>Research Ethics Board<br>P126-770 Bannatyne Avenue<br>Winnipeg, Manitoba, R3E 0W3, Canada	March 30, 2015
Canada	0010305/2	Medicine Professional Corporation<br>82 Buttonwood Ave., Ruddy Building, 3rd Floor, Suite 3-92<br>Toronto, Ontario, M6M 2J5, Canada	Dr Farooq Ismail	Western Institutional Review Board<br>1019 39th Avenue SE Suite 120<br>Puyallup, WA 98374-2115, Canada	August 1, 2014
Canada	0010089	David King, Inc.<br>303 A Herring Cove Road<br>Halifax, Nova Scotia, B3P 1M3, Canada	Dr David King	Western Institutional Review Board<br>1019 39th Avenue SE Suite 120<br>Puyallup, WA 98374-2115, Canada	September 24, 2014
USA	0010191	Mount Sanai Medical Center<br>1 Gustave Levy Place, Box 1052<br>New York, NY 10029, USA	Dr David Simpson	BRANY IRB Accreditation<br>Consulting Services<br>1981 Marcus Avenue, Suite 210<br>Lake Success, NY 11042, USA	March 2, 2015

CEC, clinical ethics committee; EC, ethics committee; LEC, local ethics committee; IRB, institutional review board.

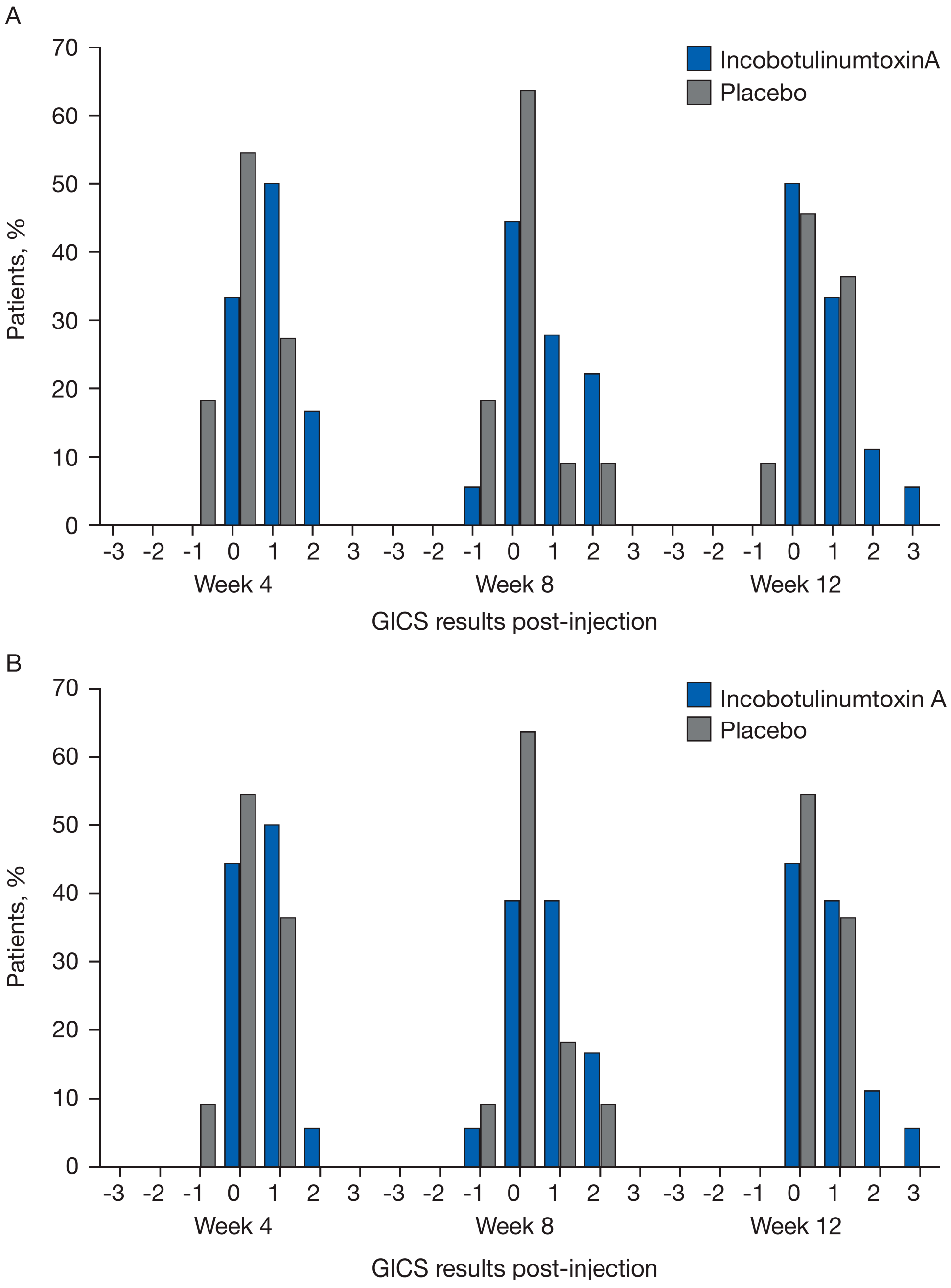

Figure S1. (**A**) Physician's and (**B**) patient's GICS at Weeks 4, 8, and 12 (full analysis set). GICS from –3 to +3; higher values indicate better results (–3, very much worse; –2, much worse; –1, minimally worse; 0, no change; +1, minimally improved; +2, much improved; +3, very much improved). GICS, global impression of change scale.

References

1. Broussolle, E.; Krack, P.; Thobois, S.; Xie-Brustolin, J.; Pollak, P.; Goetz, C.G. Contribution of Jules Froment to the study of parkinsonian rigidity. *Mov Disord* **2007**, *22*, 909–914.
2. Mendonça, D.A.; Jog, M.S. Tasks of attention augment rigidity in mild Parkinson disease. *Can. J. Neurol. Sci* **2008**, *35*, 501–505.
